# A Robotic Assistance With Specialized Timing Improves Motor Performance: Implications for Movement Training

**DOI:** 10.1109/TNSRE.2026.3660517

**Published:** 2026

**Authors:** Alex C. Dzewaltowski, Philippe Malcolm

**Affiliations:** Department of Biomechanics and the Center for Research in Human Movement Variability, University of Nebraska Omaha, Omaha, NE 68182 USA. He is now with the Dr. William M. Scholl College of Podiatric Medicine, Rosalind Franklin University of Medicine and Science, North Chicago, IL 60064 USA; Department of Biomechanics and the Center for Research in Human Movement Variability, University of Nebraska Omaha, Omaha, NE 68182 USA

**Keywords:** Robotic rehabilitation, neurorehabilitation, physical therapy, adaptation, overspeed training

## Abstract

Robotic devices can expand the repertoire of rehabilitation methods by enabling actions that cannot be replicated by a physical therapist. We previously developed a technique, we term ‘rapid assistance,’ that can assist movements beginning within the electromechanical delay between muscle activation and muscle contraction. Here, we evaluated the effects of repeated arm extension training with rapid assistance in older adults (n = 18) during a single session. We compared training with rapid assistance to a control group that performed unassisted arm extension training. Participants positively adapted to rapid assistance indicated by quickening reaction times (15.69%, *t* = −1.79, *p* = 0.089, *d* = 0.36) and greater extension angular velocities (47.93%, *t* = 3.47, *p* = 0.002, *d* = 0.56) compared to the control group following training. These motor performance improvements following rapid assistance training may be due to reducing Golgi-tendon inhibition during muscle contraction thereby, introducing an alternate strategy to improve motor performance. This specialized assistive timing may address a trade-off present in rehabilitative practice between assisting a patient or sufficiently challenging them to facilitate functional recovery.

## I. Introduction

Exercise is critical to human performance and recovery. Most rehabilitation programs rely on repeating certain exercises to improve patients’ motor function. However, there often exist limitations in patients’ ability to participate in exercise due to symptom severity [1], [2], [3], [4]. Physical therapists can modify exercises and assist patients, but they must consider a trade-off between the amount of assistance they provide and the level of challenge necessary for functional improvements. Consequently, physical therapists attempt to provide patients with just enough assistance to perform movement practice but not too much assistance to cause dependency on the assistance or prevent motor recovery [5], [6], [7], [8], [9].

Robotic devices have the potential to facilitate exercise in a similar way to physical therapists [10], [11], [12], [13], [14], [15]. Robotic rehabilitation devices have been designed to provide minimum magnitudes of assistance necessary for movement practice and do so more consistently than humans [16], [17], [18], [19]. When these robotic devices are used, patient functional improvements are similar to conventional physical therapy [9], [20]. However, robotic devices also have the potential to execute training interventions that are not replicable by a human physical therapist. By extension, robotic devices can offer new, alternative strategies of care that enhance the effectiveness of rehabilitation.

When designing robotic devices, it is established that the timing of the force applied within the movement often determines whether the effect is ‘assistive’ or not. For example, an exoskeleton designed to assist walking will not reduce metabolic cost if the timing of assistance is not appropriately synced with the movement cycle. Moreover, if the assistance is tailored to the magnitude or timing of muscle activation, the robotic device is more likely to be assistive and reduce metabolic cost [21], [22], [23], [24]. To expand on the nuanced importance of the timing of assistance relative to the movement onset, we developed a method using a hardwired EMG system that enables control of the timing of applied forces relative to the onset of muscle activation [25].

**Fig. 1.**
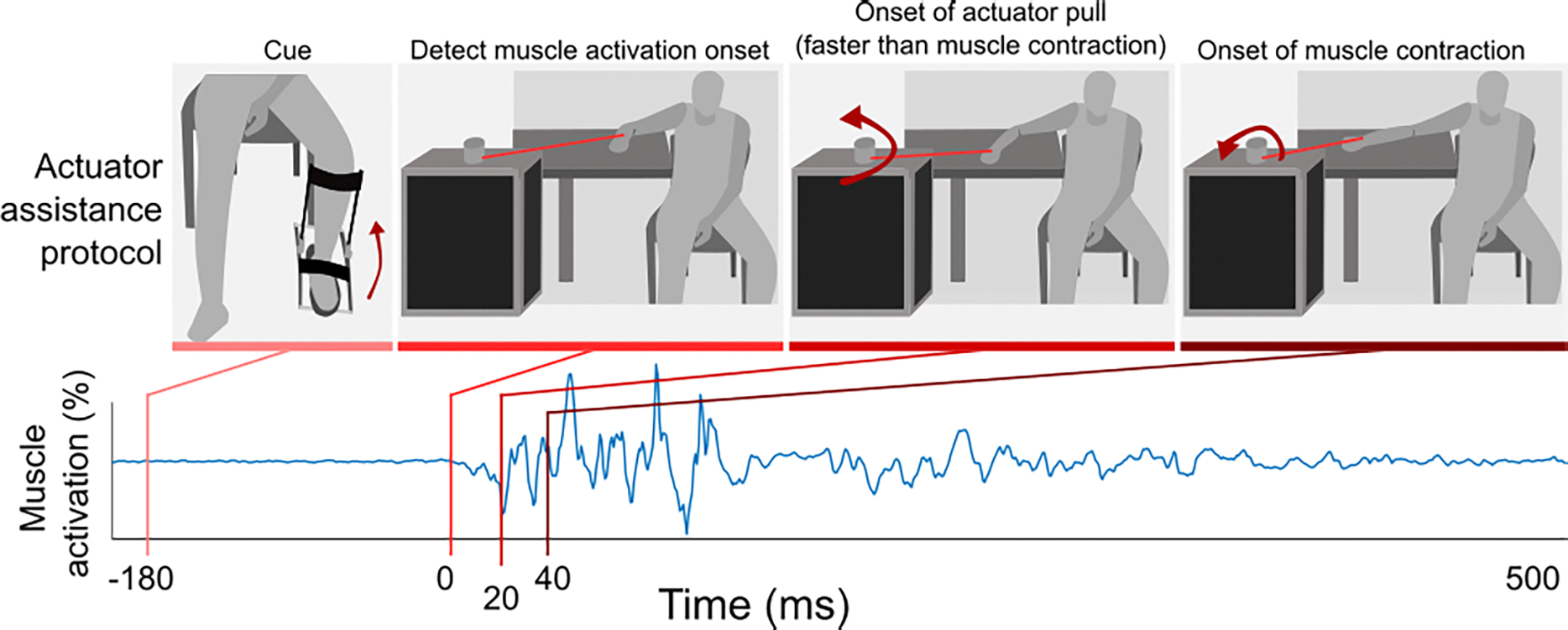
Rapid assistance condition. Participants are cued to extend their arm as quickly as possible via a ~ 2cm heel raise. Once voluntary muscle activation of the arm extensors begins, there is a ~ 40ms delay until the start of muscle contraction (i.e., the electromechanical delay). Rapid assistance is defined by detecting voluntary muscle activation and beginning tethered actuator assistance within ~ 23ms on average. This results in the participant’s arm moving prior to muscle contraction but following the participant’s voluntary neural signal to start the movement. Rapid assistance is not triggered if the participant does not decide to move in response to the cue.

We previously investigated the effect of assisting and resisting arm extension with different timings relative to the onset of muscle activation. We asked participants to extend their arms as quickly as possible once cued. The experimental setup could reliably apply assistive forces via a tethered actuator within ~23ms following the onset of muscle activation (i.e., within the electromechanical delay, Fig. 1). The electromechanical delay is the period of time (~40ms) between onset of active muscle (i.e., muscle activation) and a measured change in force (we refer to as muscle contraction) [26]. Since our setup can apply a force in ~23ms, we can move the arm beginning within the electromechanical delay and prior to the biological muscle. We showed that when robotic assistance is provided within this electromechanical delay, the assistance results in a 68.97% *increase* in the subsequent voluntary muscle activation [25]. We term this assistance within the electromechanical delay as “*rapid assistance”*.

Such a large increase in muscle activation may be useful for rehabilitation or high-intensity training (e.g., in athletes, military personnel, and first responders). However, our previous work only evaluated a short bout of actuator pulls within the electromechanical delay (30 completions) which were an insufficient number to evaluate adaptation. Generally, we expect that repetitions of a motor task should slowly reduce muscle activation over time [27], [28], [29]. A reduction in muscle activation paired with maintained performance metrics can be considered an improvement in the motor task. This leaves the question, does muscle activation progressively decrease with repetitions following its acute increase from rapid assistance and will this be paired with maintained performance metrics?

Here, we investigated rapid assistance during a single-session training intervention in an older adult cohort. Older adults exhibit slower reaction times, slower cognitive processing, and reduced motor adaptability than young adults and therefore, have greater room for improvement in motor abilities [30], [31], [32], [33], [34]. We measured muscle activation, reaction time, and peak extension angular velocity. Our primary hypothesis was that peak muscle activation of the agonist muscle would acutely increase during arm extension training with rapid assistance compared to a control group that was unassisted.

## II. Methods

We recruited 18 older adults from the greater region of Omaha, Nebraska. The University of Nebraska Medical Center’s Institutional Review Board approved the study protocol. Participants provided written informed consent prior to the study. Participants were healthy and 50–75 years old. Exclusion criteria were neurological, musculoskeletal, or cardiovascular limitations that would inhibit their ability to complete the study protocol. We randomly allocated participants to two parallel groups: nine participants were allocated to a group that performed arm extension training with rapid robotic assistance (age: 63.88 ± 7.26yrs, height: 167.25 ± 11.26cm, weight: 72.61 ± 16.04kg, females = 6) and nine participants were allocated to a control group that performed arm extension training without assistance (age: 66.50 ± 7.35yrs, height 166.88 ± 11.00, weight: 77.47 ± 16.34, females = 6). Neither the participants nor the researchers were blinded to the group they were in; however, the participants were not told about the exact aims or hypotheses of the study.

### A. Training Protocol.

**Fig. 2.**
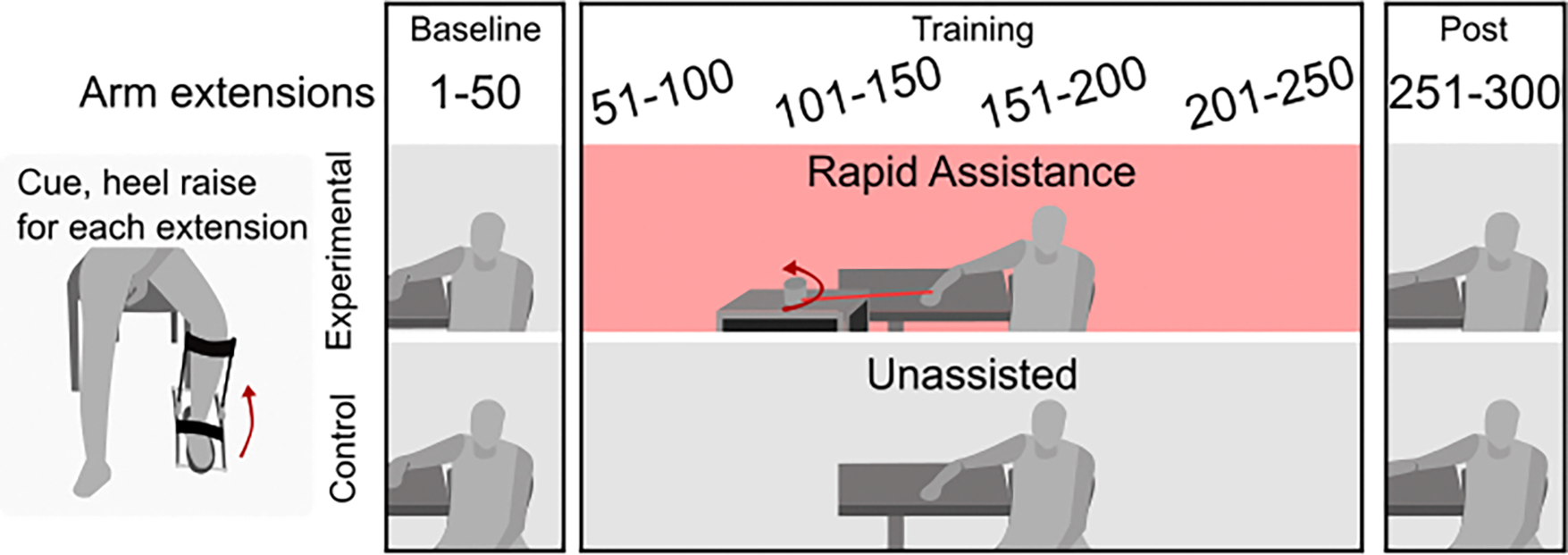
Outline of study design. Participants were divided into two groups, one that received rapid assistance and one that only performed unassisted arm extensions. Participants performed six sets of 50 arm extensions (300 total). Each arm extension was cued with a heel raise.

Each participant completed a total of 300 arm extensions, and the experimental group received rapid assistance (Fig 2). The arm extensions were divided into six sets of 50 extensions, each lasting ~13 minutes. The rest period between sets was approximately five minutes. One group received rapid assistance from the 2^nd^ to the 5^th^ set. The control group did not receive any assistance. The first (“baseline”) and last (“post”) set of 50 extensions were unassisted in both groups. Participants were instructed to complete each arm extension as quickly as possible once they felt a small push underneath their left heel. The push underneath their heel consisted of a heel raise of ~2cm and was applied using an exoskeleton end effector (HuMoTech, Pittsburgh, PA). The timing between each heel raise was randomized between 5–12s. For each arm extension, participants were seated with their arm placed on a table with a shoulder abduction angle of ~80°. Images and supplementary video of the exact setup can be found in Dzewaltowski and Malcolm (2024) [25].

The rapid robotic assistance consisted of short, assistive tethered actuator pulls with an onset time that was within the electromechanical delay between muscle activation and muscle contraction (i.e., rapid, robotic assistance). Due to being ‘tethered’ robotic assistance, participants can outpace the velocity of assistance and if they do so, the assistive force becomes zero. Surface electromyography (EMG) sensor pads were placed on the agonist muscle (triceps brachii) and recorded at 1000Hz (Noraxon, Scottsdale, AZ). A threshold of 1% above the maximum squared EMG during the previous resting period between extensions was used to detect the onset of muscle activation. Surpassing this activation threshold triggered rapid robotic assistance for one extension. The threshold was recalculated after each arm extension that would receive rapid assistance. The speed of the applied assistance was 0.8ms^−1^for 0.35s.

### B. Quantification of Training Effects.

Motion capture of the arm and hand was recorded with 16 cameras (Vicon, Oxford, UK, 200Hz). Ten retroreflective markers were placed on the shoulder, anterior and posterior lateral portions of the upper arm, the lateral epicondyle of the humerus (i.e., the elbow), the anterior and posterior lateral portions of the forearm, one placed between the radial and ulnar process (i.e., the wrist), and one placed on the head of metacarpal two, five, and three (i.e., middle finger). The angular velocity was calculated as a change in the position of the wrist about the elbow using Euler angle calculations (MATLAB 2021b). Peak magnitudes and the timing of the peak angular velocity were extracted.

To evaluate the effects of training on muscle activation, triceps, and biceps brachii EMG were rectified, high-pass filtered with a cutoff frequency of 20Hz, and then low-pass filtered with a cutoff frequency of 10Hz using a 4^th^ order Butterworth filter. Participant data were normalized against their mean peak baseline activation. Peak muscle activation of the triceps and biceps brachii for each arm extension was gathered from EMG onset to 200ms following EMG onset. EMG onset was identified after applying a Hilbert filter with a window of 10ms and a threshold calculated from the beginning, resting portion of the trial. This process was not used for real-time control but only for post-processing identification of EMG onset. Reaction time was defined as the time duration between the cue and the onset of agonist activation.

### C. Statistical Analyses.

Linear mixed models were used to test for significant effects on peak muscle activation of the triceps and biceps brachii within the first 200ms following EMG onset detection, the angular velocity of the arm’s peak magnitude, and reaction time. Two fixed effects were used for time and group and the interaction between time and group. One random effect was used for within-subject variance. Significance was evaluated at an *α*-level of 0.05. The mean was calculated for each subject for each set of 50 arm extensions for each dependent measure. The fixed effect of ‘Time’ was incremented for each set after baseline (1 to 5) corresponding to the second set of arm extensions (51–100) to the post-set of extensions (251–300). Outlier trials within the set were removed based on three standard deviations from the mean of the set. Data were then normalized against each subject’s baseline mean. Analyses were conducted in R using packages ‘lmer’ and ‘emmeans’.

## III. Results

Our primary hypothesis was that peak muscle activation of the agonist muscle would acutely increase during arm extension training with rapid assistance compared to a control group that was unassisted. We reported all measures as percentages relative to the mean during baseline, and all baseline measurement magnitudes were not significantly different between groups.

### A. Acute Increases in Voluntary Muscle Activation From Rapid Assistance

**Fig. 3.**
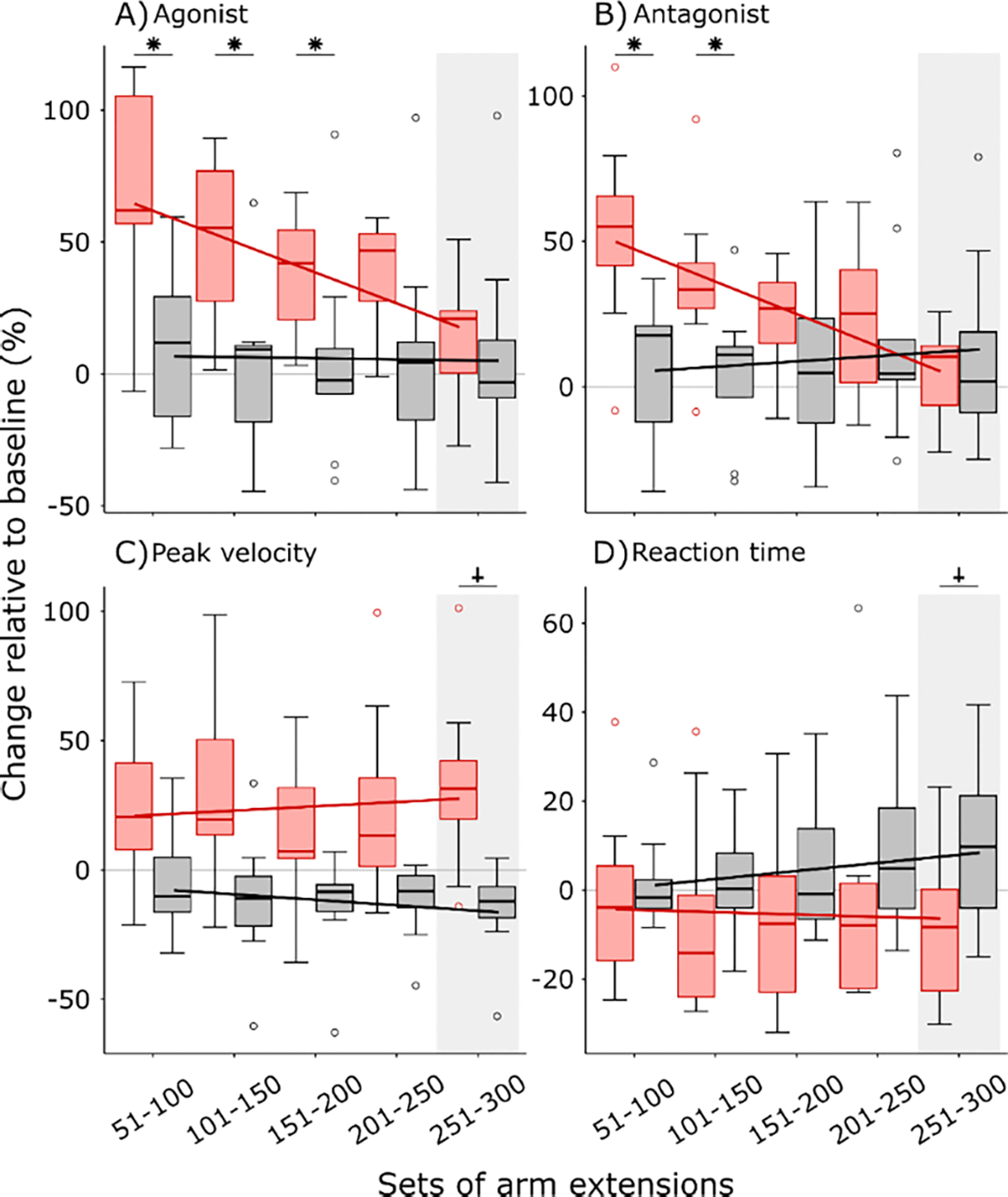
Differences between rapidly assisted and unassisted arm extension training. Data are reported as participants’ mean percent change for each set compared to their mean baseline set (repetitions 1–50). Red boxes represent rapidly assisted participants and dark grey boxes represent unassisted participants. Peak agonist activation (A), peak antagonist activation (B), peak angular velocity (C), and reaction time (D). The post-set of arm extensions (251–300), highlighted with a light grey box, are unassisted arm extensions in both groups. Box centerlines are group medians, box edges represent group interquartile range, and box whiskers represent upper and lower quartiles. Open circles are outliers that fall outside 1.5 in the interquartile range. Outlier values are not excluded from statistical analyses. Lines indicate a linear fit across group means. Rapidly assisted participants moved their arm faster following training than at their baseline with similar amounts of muscle activation. *Stars indicate significant post-hoc effects (*p* < 0.05). † Indicate trends towards siqnificance.

Participants’ peak agonist muscle activation significantly increased from initial exposure to rapid assistance but returned to baseline magnitudes over the course of the training session. We found a significant interaction between protocol time and group in peak agonist muscle activation (*SE* = 2.61, *t* = 4.32, *p* < 0.001, Fig 3.). The mean agonist peak activation of the second set of arm extensions was significantly greater (+65.81 ± 39.91%) in the group that received rapid assistance compared to the control group (+10.99 ± 28.16%; *t* = 3.61, *p* = 0.001, (Cohen’s *d* effect size) *d* = 0.563). This supports our primary hypothesis that rapid assistance would result in an acute increase in agonist activation. The magnitude of the increase in activation is similar to our previous findings in healthy, young adults (+68.97 ± 80.05%) [25]. However, the relative increase in agonist activation gradually diminished over the course of completing extensions nearing the agonist activation of the control group during the post-set. Mean agonist peak activation was not significantly different between groups for the post-set of arm extensions (*t* = 0.53, *p* = 0.600, *d* = 0.010).

Participants’ peak antagonist muscle activation significantly increased from initial exposure to rapid assistance but returned to baseline magnitudes over the course of the training session. We found a significant interaction between time and group in peak antagonist muscle activation (*SE* = 2.04, *t* = 6.35, *p* < 0.001). The effects on antagonist activation mirrored those on agonist activation. The mean antagonist peak activation of the second set of arm extensions was significantly greater (+52.19 ± 33.18%) in the group that received rapid assistance compared to the control group (+6.09 ± 24.73%; *t* = 3.52, *p* = 0.002, *d* = 0.568). The increase in antagonist activation due to rapid assistance is in contrast to the results of our previous study in healthy, younger adults, which did not increase antagonist activation [25]. This finding may be attributed to older adults coactivating muscles more than younger adults [28], [35], [36]. Antagonist activation diminished over the course of completing arm extensions and returned to baseline activation magnitudes. Mean antagonist peak activation was not significantly different between groups for the post-set of arm extensions (*t* = −0.56, *p* = 0.581, *d* = 0.109).

### B. Post-Training Improvements to Motor Performance From Rapid Assistance

Rapid assistance led to slightly decreased reaction times whereas the control group had slightly increased reaction times following the training session. We found a significant interaction between time and group in reaction time (*SE* = 1.09, *t* = 2.14, *p* = 0.036). The reaction time of the post-set of arm extensions for the group that received rapid assistance trended toward being significantly faster than the control group (*t* = −1.79, *p* = 0.089, *d* = 0.36). Reaction times during the post-set arm extensions were faster for rapidly assisted participants (−7.15 ± 16.81%), whereas unassisted participants had slower reaction times (+8.54 ± 18.51%). Faster reaction times is an effect that precedes rapid assistance and persists after cessation of rapid assistance during the post-set of arm extensions. Therefore, the human-robotic interaction from rapid assistance has effects outside its specific temporal application.

Participants who received rapid assistance consistently moved their arm faster than the control group. No significant main effects were found in the peak angular velocity of the arm across time (*SE* = 1.67, *t* = −1.78, *p* = 0.305), but there was a trend toward significance between groups (*SE* = 14.01, *t* = −1.78, *p* = 0.087). We consider this potential difference between groups reasonable to assess post-hoc for the post-set comparison due to an expected differential effect on arm extension velocity from the cessation of rapid assistance. With the cessation of rapid assistance, the group that received rapid assistance had faster peak arm extension velocity (+32 ± 34.19%) than the control group (−15.93 ± 18.64%) during the post-set (*t* = 3.47, *p* = 0.002, *d* = 0.56). Additional assessments of angular velocity can be found in supplementary materials. Importantly, participants greatly outpaced rapid assistance on average meaning the peak angular velocities reported in Fig 3 are achieved by voluntary arm movement (Fig S1). Furthermore, these arm extension velocities are maintained throughout the final post-set (Fig S2).

## IV. Discussion

We evaluated the effects of rapid assistance during a single training session composed of repeated arm extensions. Based on our previous study in younger adults, we hypothesized that rapid assistance would acutely increase muscle activation of the agonist (triceps brachii) compared to repeated arm extensions without assistance. We also hypothesized that this increase would diminish over time. Our findings support that older adults adapt over time to rapid assistance, where rapid assistance has the potential to improve motor performance. This lays a foundation for assistive forces applied within the electromechanical delay to be useful in motor rehabilitation.

In our previous work, we discussed several potential explanations for the large, acute increase in agonist activation from rapid assistance, such as the superimposition of sensory information, eliciting of a startle reflex, and reduction in Golgi tendon inhibitory signaling. Here, we replicated the acute increase in muscle activation of ~65% in older adults. Similar to our previous work in young adults, we conclude this magnitude is unlikely to be attributed to a summation of a superimposition of sensory afference with voluntary muscle activation. Alternatively, previous work from other researchers has applied pressure or vibration to the sole of the foot, which attenuates subsequent magnitudes of voluntary muscle activation of the soleus and tibialis anterior [37], [38], [39], [40]. The superimposition of sensory afference may adjust key excitation thresholds for voluntary activation, which, in turn, can increase activation magnitudes. However, this kind of effect does not seem likely to result from rapid assistance. Rapid assistance physically moves the arm beginning ~23ms following the onset of voluntary muscle activation, which is very different from statically applying pressure or vibration prior to voluntary muscle activation. Due to the direct interaction with the movement and the narrow time window, it is unlikely that the superimposition of sensory afference is the primary driver for the acute increase in muscle activation from rapid assistance.

We considered the release of a preplanned movement via a startle reflex as another potential explanation for the acute increase in muscle activation [41], [42], [43]. A startle reflex can consistently increase muscle activation compared to a solely volitional movement and could potentially explain the acute increase in muscle activation due to rapid assistance. If rapid assistance is a startling stimulus, the reduction in activation we observed over time would likely indicate habituation rather than motor adaptation [44], [45], [46]. However, we also found a reduction in reaction time and elevated arm extension velocity as muscle activation decreased over the course of training with persistence through the post-condition. Habituation would not correspond to positive effects on motor performance, and therefore, a startle reflex is insufficient to explain our findings.

Based on elimination, our leading explanation for the effect of rapid assistance is a decrease in Golgi tendon organ inhibition [47], [48]. Rapid assistance may decrease muscle tension due to faster actuation and, as a consequence, reduce the amount of inhibitory signaling from Golgi tendon organs. The decrease in inhibitory signaling explains the initial, substantial increase in agonist activation. Moreover, the reduction in activation over many repetitions may be an adaptive response to excess muscle activation. A training intervention that reduces the amount of inhibitory signaling may support the conditioning of faster motor function. In addition, this explanation is further supported by the reduction in antagonist activation, the trend toward a faster reaction time, and the maintenance of an elevated arm extension peak angular velocity.

We have focused on sensory and spinal mechanisms to explain our observed findings from rapid assistance, however, areas of the brain may still contribute to the adaptation observed. We suspect Golgi tendon inhibition is reduced due to the application of force from rapid assistance, but the changes observed from repetitions may also point to cortical mediation of muscle activation. Reciprocal inhibition refers to the suppression of antagonist muscle activity during movement and is initiated by cortical as well as spinal regions [49], [50], [51]. Reciprocal inhibition more generally refers to coordination between agonist and antagonist muscles where the objective is to decrease overall muscle activation to efficiently perform the movement. Explosive strength training has been shown to increase reciprocal inhibition; focusing on decreases to antagonist muscle activation [49], [52]. Rapid assistance may present similarities to explosive strength training and thereby, facilitate reciprocal inhibition (via central and/or peripheral pathways).

The reduction in muscle activation over time aligns with previous findings of adaptation resulting from repeated practice [53], [54], [55], [56]. However, other explanations for the progressive reduction in muscle activation may be due to either adaptation, a decrease in arm extension velocity, reduced co-contraction from familiarization, or reduced effort. Our conjecture is that the progressive decrease in muscle activation during rapid assistance training is due to adaptation because they are coupled with faster angular velocities throughout and following training. Moreover, there was also a trend toward faster reaction times in the rapid assistance group (−7.15 ± 16.81%), compared to a slight increase in reaction time in the control group (+8.54 ± 18.51%) further supporting participants adapted to rapid assistance. Although the reaction time reduction was small, it is meaningful given the simplicity of the task, suggesting that rapid assistance could have rehabilitation potential. However, the trend of increase in reaction time in the control group could be attributed to boredom [57]. Boredom has been reported to negatively affect participants’ reaction times as well as increase their mental fatigue despite lower amounts of self-reported effort [58].

Concerning broader applications, we posit that the adaptive timing of the assistance may be critical to our initial finding that peak voluntary muscle activation increases. A consistent robotic assistive force that moves quicker than the participant’s muscle may present a training paradigm akin to overspeed training. Over-speed training is predominately used by athletes who believe it improves motor coordination and speed at performing a particular movement. Over-speed training can take the form of downhill running, decreasing the standard weight of a ball being thrown, or the use of other types of assistive equipment that allows the body to move faster than it would otherwise be able [59], [60], [61]. While overspeed training seems somewhat commonplace in exercise programs, its effects have not yet been clearly separated from those of other standard resistive exercises. This work presents a mechanism that may offer an opportunity to refine approaches to overspeed training by emphasizing a decrease in inhibitory signaling.

Rapid assistance may present an alternative exercise that challenges neurological control speeds rather than muscle strength or cardiovascular endurance. Other exercise paradigms exist which ‘challenge neurological control’ rather than focus on muscle development such as split-belt treadmill walking, robotic force fields for upper limb rehabilitation in patients, and perturbation-based balance training in patients with neurological pathologies such as stroke, multiple sclerosis and Parkinson’s Disease [9], [10], [12], [62], [63]. While it is difficult to predict the effects of rapid assistance in any neurological pathology at this stage, our findings suggest the rapid assistance offers a training paradigm that can target specific muscle groups to improve rapid movement performance.

## V. Conclusion

Rapid assistance initially increased muscle activation, but this increase diminished as participants adapted over repeated exposure with no increase following training. This adaptation to rapid assistance seems to positively affect motor function, as supported by a decreased reaction time (15.69%) and sustained, elevated peak arm extension angular velocity (47.93%) compared to the control group following training. The adaptive timing of rapid assistance that is *just* quicker than the biological muscle may address fundamental tradeoffs between providing assistance and sufficiently challenging patients during physical therapy and rehabilitation. Moreover, our initial pilot work involving one participant with multiple sclerosis with upper-limb, non-resting tremor offers a proof-of-concept for implementation of rapid assistance in complex, neurological pathology [64]. Rapid assistance may access a novel neural mechanism for improving motor performance, and its implementation presents a consistent, graded exercise challenge that inherently shifts the difficulty level with participant capability. Future work will look to investigate the effect of rapid assistance following multiple training sessions in addition to expanding the number of trainable movements.

## Supplementary Material

tnsre-3660517-mm

This article has supplementary downloadable material available at https://doi.org/10.1109/TNSRE.2026.3660517, provided by the authors.
