# Supplementary material for: A Robotic Assistance With Specialized Timing Improves Motor Performance: Implications for Movement Training
Source: IEEE Trans Neural Syst Rehabil Eng. Author manuscript; Available in PMC 2026 Apr 1. (PMC13042254; doi:10.1109/TNSRE.2026.3660517)
Supplement: tnsre-3660517-mm [file NIHMS2150614-supplement-tnsre-3660517-mm.zip › tnsre-3660517-mm/2026-01-16_Supplementary_FastBioTSNRE.pdf]

Supplementary material.

We only approximated the applied angular velocity during rapid assistance because we did not have measures of where the actuator was relative to the participant's arm. However, to provide some reference, if we assume the $0.8ms^{-1}$ pull from the actuator is applied perfectly tangential to the arm rotation (it is not perfectly tangential for the majority of the extension) and assume the arm is a length of 30cm (average distance from elbow to palm), the peak applied angular velocity is $2.7rads^{-1}$. This idealized peak applied angular velocity is meaningfully larger than our actual application throughout arm extension. Seen in Fig S1, most participants have angular velocities well above this cutoff, meaning participants generally outpaced the assistance provided. By extension, the percent change in angular velocity reported in Figure 3 of the main text is representative of voluntary arm movement. In addition, the increase in angular velocity during the post-set is reasonably consistent across trial completions.

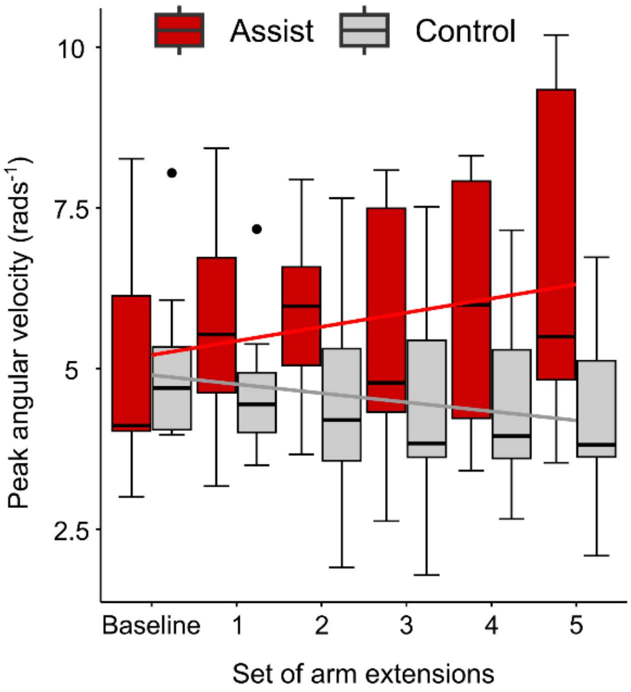

Fig S1. Measured peak angular velocity of arm extension. Data are participant mean magnitudes of arm extension for each set. Set 5 corresponds to the post-set of arm extensions for each group. We estimate the angular velocity of rapid assistance to be less than $2.7rads^{-1}$ (meaningful variation is present due to anthropometrics). This illustrates that participants generally outpaced rapid assistance, meaning the measured peak angular velocity is from participant ability. As a reminder, rapid assistance is applied via a tether in tension. Therefore, the assistive force provided immediately becomes zero when participants outpace rapid assistance.

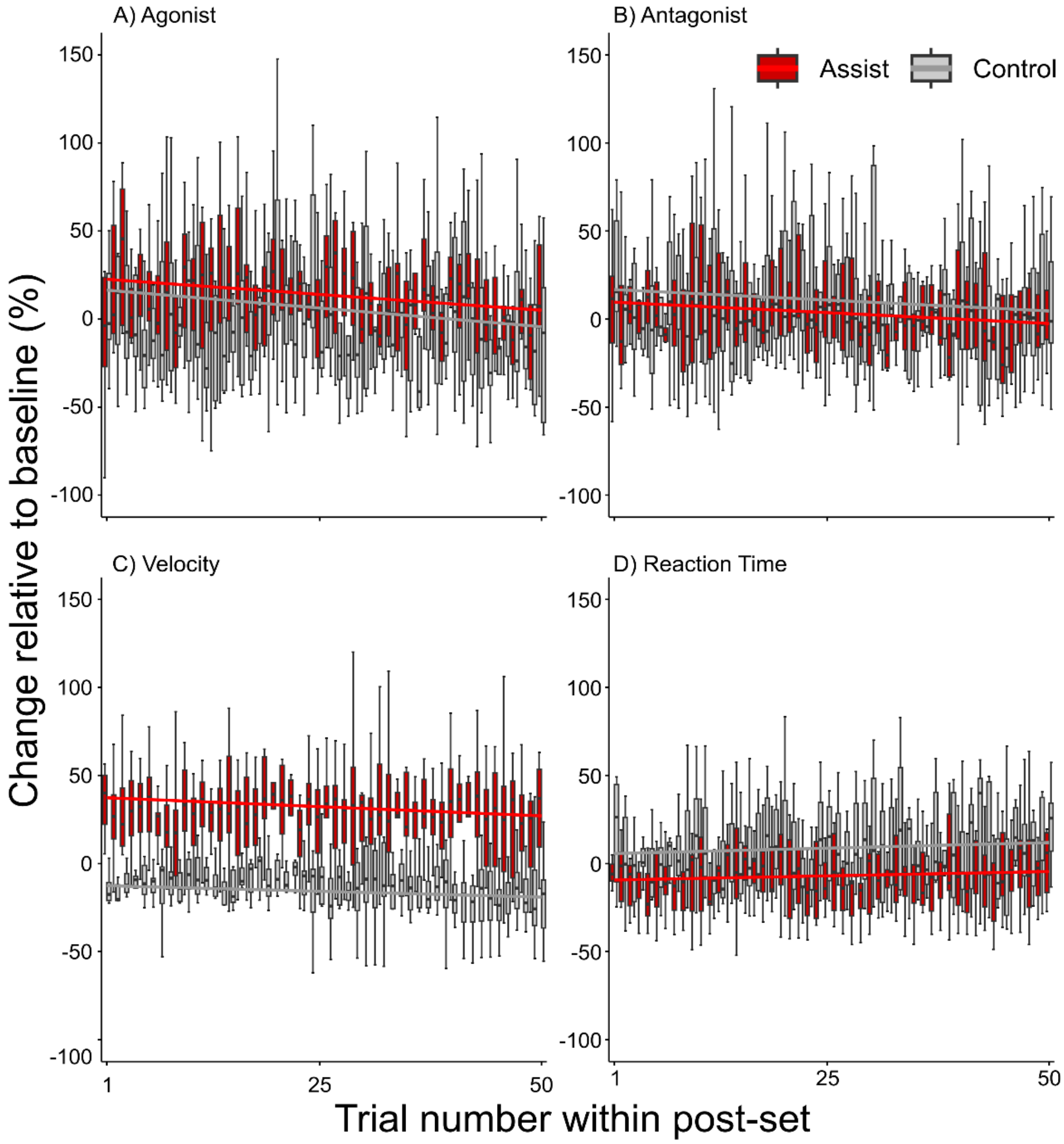

Fig S2. Percent change relative to baseline during the post-set of arm extensions. Plots are of every arm extension completed by every participant. Lines are linear fits against the mean values across all participants for each trial (1-50) completed in the post-set.

In the assist group, 80.2% of the trials completed during the post-set (361 out of 450) were faster velocities than baseline. In the control group, 26.6% of the trials completed during the post-set (133 out of 450) were faster velocities than baseline.

	Group	Slope	Intercept	Extrapolated trials
**Agonist**	Assist	-0.358	23	15
	Control	-0.423	17	-9
**Antagonist**	Assist	-0.247	10	-9
	Control	-0.248	17	19
**Velocity**	Assist	-0.210	38	131
	Control	-0.132	-12	N/A
**Reaction Time**	Assist	0.097	-9.5	48
	Control	0.127	6	N/A

Table S1. The linear equation to the trends plotted on Figure S2. Column, '*Slope'*, is the linear coefficient between the percent change relative to baseline and number of trials completed. Column, *Intercept*, represents the percent change at zero trials completed in the post-set. Column, *Extrapolated trials,* indicate the additional number of trials needed for the linear trends to return to baseline magnitudes beyond the post-set. *Extrapolated trials* that are negative are group measures that returned to baseline during the post-set of 50 trials. Values labeled as 'N/A' identify linear trends that already achieved baseline levels prior to beginning the post-set.

The purpose of Figure S2 and Table S1 is to clarify the consistency in participant performance within the post-set of arm extensions, and to establish an estimate for the number of trials post-training required for washout.
